# Past, present, and future of allergen immunotherapy vaccines

**DOI:** 10.1111/all.14300

**Published:** 2020-04-29

**Authors:** Yulia Dorofeeva, Igor Shilovskiy, Inna Tulaeva, Margarete Focke‐Tejkl, Sabine Flicker, Dmitriy Kudlay, Musa Khaitov, Antonina Karsonova, Ksenja Riabova, Alexander Karaulov, Roman Khanferyan, Winfried F. Pickl, Thomas Wekerle, Rudolf Valenta

**Affiliations:** ^1^ Division of Immunopathology Department of Pathophysiology and Allergy Research Center for Pathophysiology, Infectiology and Immunology Medical University of Vienna Vienna Austria; ^2^ National Research Center, Institute of immunology, FMBA of Russia Moscow Russian Federation; ^3^ Department of Clinical Immunology and Allergy Laboratory of Immunopathology Sechenov First Moscow State Medical University Moscow Russian Federation; ^4^ Department of Immunology and Allergy Russian People’s Friendship University Moscow Russian Federation; ^5^ Institute of Immunology Center for Pathophysiology, Infectiology and Immunology Medical University of Vienna Vienna Austria; ^6^ Section of Transplantation Immunology Department of Surgery Medical University of Vienna Vienna Austria

**Keywords:** allergen, allergen‐specific immunotherapy, allergy, molecular allergy vaccines

## Abstract

Allergen‐specific immunotherapy (AIT) is an allergen‐specific form of treatment for patients suffering from immunoglobulin E (IgE)‐associated allergy; the most common and important immunologically mediated hypersensitivity disease. AIT is based on the administration of the disease‐causing allergen with the goal to induce a protective immunity consisting of allergen‐specific blocking IgG antibodies and alterations of the cellular immune response so that the patient can tolerate allergen contact. Major advantages of AIT over all other existing treatments for allergy are that AIT induces a long‐lasting protection and prevents the progression of disease to severe manifestations. AIT is cost effective because it uses the patient´s own immune system for protection and potentially can be used as a preventive treatment. However, broad application of AIT is limited by mainly technical issues such as the quality of allergen preparations and the risk of inducing side effects which results in extremely cumbersome treatment schedules reducing patient´s compliance. In this article we review progress in AIT made from its beginning and provide an overview of the state of the art, the needs for further development, and possible technical solutions available through molecular allergology. Finally, we consider visions for AIT development towards prophylactic application.

**Abbreviations**
AEs: Adverse events
AIT: Allergen‐specific immunotherapy
Breg cells: B regulatory cells
DC: Dendritic cell
ELISA: Enzyme‐linked immunosorbent assay
EPIT: Epicutaneous immunotherapy
GMP: Good Manufacturing Practice
IgE: Immunoglobulin E
IgG: Immunoglobulin G
ILIT: Intralymphatic immunotherapy
MPL: Monophosphoryl lipid A
ODN: oligodeoxynucleotide
OIT: Oral immunotherapy
PBMC: Peripheral blood mononuclear cells
pDC: plasmacytoid dendritic cell
PEG: Polyethylenglycol
SCIT: Subcutaneous immunotherapy
SLIT: Sublingual immunotherapy
SPT: Skin prick test
TFH cells: T follicular helper cells
TFR cells: T follicular regulatory cells
TH: T helper
TLR: Toll‐like receptor
VIT: Venom immunotherapy
VLP: Virus‐like particle
VNP: Virus‐like nanoparticle

## 1 INTRODUCTION

IgE‐associated allergy, the most common immunologically mediated hypersensitivity disease, is based on the formation of IgE antibodies against per se harmless and mainly environmental antigens, termed allergens.^1^ Subjects with a genetic predisposition for allergy (ie, atopic subjects) produce IgE antibodies against allergens in their environment.^2^ IgE binds to mast cells and basophils via high‐affinity receptors for IgE so that subsequent allergen contact can induce mast cell and basophil activation by cross‐linking of cell‐bound IgE. This leads to release of inflammatory mediators and cytokines and thus immediate allergic inflammation.^3^, ^4^ Antigen‐presenting cells, especially B cells and dendritic cells can bind IgE via the low‐ or high‐affinity receptor for IgE, and via IgE‐facilitated allergen presentation cause T‐cell activation and secretion of inflammatory Th2 cytokines leading to activation of eosinophils and formation of innate Th2‐like immune cells such as group 2 innate lymphoid cells (ILC2s).^5^, ^6^ In contrast to allergic patients, nonallergic subjects produce allergen‐specific IgG antibodies without experiencing allergic inflammation upon allergen contact.^7^, ^8^ While anti‐inflammatory treatment based on pharmacotherapy and biologics which neutralize allergen‐specific IgE or inflammatory cytokines can ameliorate allergic inflammation, only AIT represents a causative treatment.^9^ In fact, AIT induces a protective immunity in allergic patients consisting of allergen‐specific IgG antibodies which serve as “blocking antibodies”. They prevent IgE from binding to the allergens and thus the complete consecutive downstream cascade of allergic inflammation induced by IgE allergen immune complexes.^10^, ^11^ AIT also profoundly affects allergen‐specific cellular responses which may be also due to the effects of blocking IgG antibodies and direct, not yet fully understood, effects on cells of the adaptive and innate immune system such as allergen‐specific Treg cells and other immune regulatory components.

Cell types receiving current attention in the context of AIT are T follicular helper cells, follicular regulatory T cells, and B regulatory cells. T follicular helper (TFH) cells are defined by the CXCR5 surface receptor and help in B‐cell maturation and immunoglobulin class switching. CXCR5+FoxP3+ Treg cells are a subset of Tregs, called follicular regulatory T (TFR) cells, which are capable of suppressing T‐ and B‐cell responses by migrating to germinal centers of lymph nodes.^12^, ^13^

A study by grass pollen immunotherapy has shown a significant decrease in memory TFH cell numbers after immunotherapy.^14^ Additionally, TFR cells were found to produce more IL‐10 compared with TFH cells. A possible plasticity between TFH and TFR cells has been suggested in the same study, indicating that TFR cells may play important roles in suppressing TH2 responses during AIT.^14^

IL‐10‐secreting allergen‐specific Breg cells which may be capable of suppressing allergen‐specific CD4 + T cells and producing allergen‐specific IgG4 antibodies have been identified in bee venom–tolerant beekeepers and patients having received venom AIT^15^ as well as in house dust mite allergen immunotherapy.^16^

Additionally, Breg cells may have inhibitory capacity by producing IL‐35 and TGF‐β.^17^ Apart from Treg and Breg cells, IL‐10‐secreting natural killer regulatory cells have also been shown to suppress allergen‐stimulated T‐cell proliferation in humans and may be important in tolerance induction as other regulatory cell types.^18^ All these aspects and their relevance for AIT are currently being investigated.

Major advantages of AIT are that, conceptually, AIT is a therapeutic vaccination which induces a protective allergen‐specific immune response. Only small amounts of the disease‐causing allergen or allergen derivatives are needed for generating and maintaining the protective immune response. Like other vaccination approaches, costs for treatment are affordable.^19^ Usually, after 3 years of AIT treatment beneficial effects continue for a few years, even when AIT has been discontinued.^20^ These long‐term effects may be attributed to the persistence of high‐affinity and functional allergen‐specific IgG_4_ antibodies.^21^, ^22^, ^23^ However, boosting of antibody responses may become necessary after discontinuation. One of the most important aspects of AIT is that it seems to halt the progression of mild symptoms toward severe symptoms as has been demonstrated in children.^24^ In fact AIT, but not pharmacological treatment prevented the progression of allergic rhinitis toward asthma.

However, there are also some major problems which prevent the broad application of AIT.^25^ First of all, AIT is a form of precision medicine which requires that the disease‐causing allergens are identified and the correct vaccine is administered. Second, polysensitization against many different allergens requires that for each of these allergens effective and safe vaccines are available and can be co‐administered. Third, administration of allergen to patients by AIT can cause side effects. Therefore, very cumbersome treatment schedules starting with tiny doses up to maintenance doses and multiple administrations make the treatment inconvenient for patients leading to low patients' compliance. Finally, the quality of natural allergen extracts represents a major bottle neck for producing safe and effective AIT vaccines.^26^ Additional issues are to define the optimal time to start AIT, dosing as well as intervals, when to stop/continue AIT, and there is a need for biomarkers. Therefore, we will review briefly some important milestones in the evolution of AIT before we discuss current AIT and its unmet needs.

## 2 THE IMPORTANCE OF THE PAST OF AIT

The famous quotation of Confucius, the Chinese philosopher and reformer (551 BC‐479 BC), “Study the past if you would define the future” indeed applies very much for AIT because one will then realize that important discoveries have been made long ago and that a continuous improvement along these milestones will allow us not only to improve AIT but eventually also to use the AIT treatment concept for specific prevention of allergy. Therefore, we will have a look into the historic development of AIT.

### 2.1 Some milestones in the development of AIT

In 1911, when Leonard Noon published the first study showing that injection of pollen extract improved symptoms of grass pollen allergy, the immunological basis of hypersensitivity to grass pollen was not known.^27^ At that time, neither IgE antibodies nor allergens had been characterized and the assumption was that pollen contained a toxic substance which is responsible for the inflammatory reaction. The obvious question was why Noon considered active immunization with a “toxic” component which per se would induce a toxic reaction. The answer can be found in Noon's original paper where he refers to earlier work published by Dunbar in 1903 who had shown that antisera raised in animals against pollen toxin could neutralize this “pollen toxin”.^28^ Thus, Noon considered that it may be possible to induce an “anti‐pollen toxin” immune response also by active immunization. In Table 1 we have listed some of the important milestones in AIT in a chronologic order and provide for each of those the corresponding references.

**TABLE 1:** Some milestones in the development of AIT

1903	Pollen‐specific antisera from immunized animals protect allergic patients from reactions	Dunbar^28^
1911	First desensitization with grass pollen extract	Noon^27^
1913	Vaccination by ragweed pollen extract	Clowes^29^
1921	Definition of components required for the development of an allergic reaction	Prausnitz and Küstner^30^
1927	First OIT attempt with pollen extract	Black^54^
1935	Suppression of allergen‐specific skin reactivity by post‐SCIT sera	Cooke et al^33^
1938	First AIT with Aluminum hydroxide‐adsorbed allergen extracts	Sledge^38^
1940	Isolation and characterization of allergen‐specific blocking IgG antibodies	Loveless et al^34^
1954	First double‐blind, placebo‐controlled AIT trial	Frankland and Augustin^58^
1966 1967	Discovery of IgE antibodies	Ishizaka et al^31^ Johansson and Bennich^32^
1968	AIT long‐term trial showing dose‐effect of allergen mix and asthma reduction in children	Johnstone^59^
1964 1969 1977 1981	Modified allergen extracts with low allergenic activity (haptens, PEG modified, and allergoids)	Malley et al^39^ Attallah and Sehon^40^ Lee and Sehon^41^ Marsh et al^42^
1976	Treatment of ragweed allergy by passive immunization with hyper gamma immunoglobulin	Rubinstein et al^35^
1981	AIT with allergoids	Norman et al^43^
1986	Low‐dose SLIT for dust mite allergy	Scadding and Brostoff^55^
1996	First AIT with synthetic allergen‐derived T‐cell peptides	Norman et al^44^ Simons et al^45^
1996	Plasmid DNA vaccination in mice	Raz et al^48^ Hsu et al^49^
1999	Demonstration of long‐term effects of AIT after discontinuation	Durham et al^61^
2002	Demonstration that AIT prevents the progression of allergic rhinitis to asthma	Möller et al^60^
2004	First AIT trial with recombinant hypoallergenic derivatives	Niederberger et al^62^
2005 2008	First AIT trials with recombinant wild‐type allergens	Jutel et al^46^ Pauli et al^47^
2006	AIT with Amb a 1 conjugated to a TLR 9 agonist	Creticos et al^51^
2012	Intralymphatic AIT with purified recombinant Fel d 1 hypoallergens	Senti et al^57^
2015 2016	First clinical safety and AIT studies with recombinant B‐cell epitope‐based grass pollen allergy vaccines	Niederberger et al^52^ Zieglmayer et al^53^
2017	First clinical AIT study with a plasmid DNA vaccine	Su et al^50^
2017 2018	First clinical study with recombinant allergen‐specific human IgG antibodies for passive immunization	Durham et al^37^ Orengo et al^36^

It thus becomes clear that the first AIT studies including the ragweed SCIT study by Clowes^29^ were performed before fundamental mechanisms of immediate‐type hypersensitivity were established. The study by Prausnitz & Küstner in 1921^30^ was important because it demonstrated that immediate‐type hypersensitivity can be transferred by a serum factor which then was defined as reagin (ie, IgE antibodies) and was specific for a certain antigen (ie, allergen). In addition, a tissue component which is present in allergic as well as nonallergic subjects (ie, mast cells) was needed for an immediate‐type allergic reaction. The study by Prausnitz & Küstner was also important because it provided researchers with an experimental system (ie, Prausnitz‐Küstner reaction) which could be used to search for allergen‐specific sensitization by in vivo testing and opened the possibility to identify IgE antibodies as the key serum factors in allergic reactions.^31^, ^32^ Key topics in AIT were the following:

First, there were several mechanistic studies which indicated that the induction of allergen‐specific IgG blocking antibodies is an important mechanism in AIT. The study by Dunbar conducted in 1903 demonstrated that allergic reactions can be specifically prevented with anti‐sera raised against allergen preparations already before the first AIT study was conducted by Noon.^28^ In 1935 Cooke published a seminal paper showing that allergen‐specific skin reactivity can be suppressed by post‐AIT sera using the Prausnitz‐Küstner reaction in human subjects.^33^ Loveless then identified allergen‐specific IgG antibodies as the serum factor responsible for the suppression of allergic reactions.^34^ The original experiment conducted by Dunbar was confirmed by the demonstration that allergy can be also treated with human hyper gamma immunoglobulin.^35^ Finally, a recent study demonstrated that immediate symptoms of cat allergy can be prevented by the administration of recombinant human IgG_4_ antibodies specific for the major cat allergen, Fel d 1, confirming that blocking IgG antibodies are of key importance for suppression of allergic symptoms.^36^, ^37^

Second, major attempts were made to improve the quality and specificity of AIT as well as the safety of AIT by decreasing side effects. In this context the study by Sledge needs to be mentioned which demonstrated that the adsorption of allergens onto the adjuvant Aluminum hydroxide prevented the systemic release of allergens in the body and thus reduced severe systemic anaphylactic side effects.^38^ It was then noted that allergen fragments obtained by isolating low molecular weight material from allergen extracts or by digestion exhibited reduced allergenic activity.^39^, ^40^ Furthermore, attempts were made to reduce the allergenic activity of allergen preparations by chemical modification such as conjugation to polyethylenglycol (PEG)^41^ or denaturation yielding allergoids.^42^, ^43^ With the availability of defined allergen sequences and structures in the era of molecular allergology, allergen‐derived synthetic peptides containing T‐cell epitopes without IgE reactivity were prepared as well as recombinant allergen derivatives with reduced allergenic activity and used in the first AIT trials.^44^, ^45^ Conceptually, it was important to demonstrate that recombinant allergen molecules can replace complex allergen extracts for AIT as was demonstrated in the first AIT studies performed with purified recombinant birch pollen allergen, Bet v 1, and recombinant timothy grass pollen allergens.^46^, ^47^

Also plasmid DNA vaccination was tested in experimental animal models and has been used in a first AIT trial recently.^48^, ^49^, ^50^ The conjugation of allergens with immunomodulatory DNA sequences represented another strategy to reduce the allergenic activity of the AIT vaccine and to have immunomodulatory function.^51^ Carrier‐protein bound allergen‐derived B‐cell epitope peptides represent the newest generation of recombinant hypoallergenic allergen derivatives which have entered clinical trials.^52^, ^53^ The grass pollen allergy vaccine BM32 was not only found to be nonallergenic but also induced strong allergen‐specific IgG responses. It also did not boost allergen‐specific IgE production and hence may be considered for prophylactic vaccination.^53^

In addition to modifications in AIT vaccines to be more specific and safe, different application routes have been explored with a view of making AIT more convenient. Early oral immunotherapy (OIT) dates back at least to 1927 and currently is tested for various forms of food allergy.^54^ Sublingual immunotherapy (SLIT) started in 1986^55^ and in 1998 Passalacqua conducted the first randomized controlled SLIT study with allergoid.^56^ Intralymphatic AIT (ILIT) was tried in patients even with recombinant allergen derivatives.^57^ Regarding AIT trial design and indications for AIT, the first double‐blind, placebo‐controlled AIT trial by Frankland and Agustin should be mentioned^58^ which was very important because there is a significant placebo effect in AIT. Positive effects of AIT for the treatment of asthma^59^ and the prevention of the progression of rhinitis towards asthma^60^ are also very noteworthy. Likewise, the demonstration of long‐term effects of AIT after discontinuation should be mentioned.^61^ The first AIT study with recombinant hypoallergenic allergen molecules was published in 2004.^62^

## 3 CURRENT SITUATION OF AIT

### 3.1 Can allergen extract‐based AIT be improved?

All of the AIT vaccines which are available today are based on allergen extracts which are obtained from natural allergen sources. There are huge differences among allergy vaccines from different companies and also variations from one batch to another batch produced by a given manufacturer depending on the quality and purity of the raw materials, the methods of extraction and the representation of the individual allergen molecules and their immunogenicity.^26^ Today most drugs and biologics (eg, blood coagulation factors, protein/peptide hormones, cytokines) are produced by recombinant expression, partly because of low abundance in natural sources, and purification of natural components belongs to the past. However, AIT vaccines are still produced from natural allergen sources, although today almost all relevant allergen molecules can be easily produced by recombinant expression.^25^, ^63^ In the meantime, multiple studies document that the quality of natural allergen extracts (eg, grass pollen, birch pollen, house dust mites, dog, *Alternaria*) is poor.^64^, ^65^, ^66^, ^67^, ^68^, ^69^ For example, it has been shown that important allergen molecules are lacking in certain extracts, that the ratios of different allergens vary greatly, and that natural extracts can be contaminated with unwanted materials.^70^ The molecular analysis of allergen‐specific immune responses of patients undergoing AIT with natural allergen extracts has revealed that protective IgG antibodies are not induced against all important allergens.^71^ It was shown that IgE sensitizations can be induced against allergens against which the patient was not sensitized before^72^ and that incomplete induction of protective IgG responses is associated with less favorable clinical outcome.^73^

The analysis of availabilities of AIT vaccines which have been evaluated in clinical studies sufficiently enough to provide evidence for efficacy and to follow the current rules for registered drugs has shown that only few AIT vaccines fulfill the current requirements for drugs.^26^ The majority of AIT vaccines will therefore only be available on a named patient´s basis without the current documentation of efficacy and safety required for registered drugs. Attempts to obtain registration for allergen extract‐based AIT vaccines by conducting the necessary large clinical trials up to phase III have been often not successful. One recent example is the failure of a subcutaneous birch pollen allergy vaccine in a phase III study^74^ which did not reach its endpoints.

The limitations of allergen extract‐based technologies are also evidenced by a “new” SCIT approach which is based on an extract from *Lolium perenne* pollen which was then hydrolyzed to reduce its allergenic activity. The hydrolyzed extract containing peptides of a size between 1 and 10 kDa was then used for SCIT without any adjuvant. In the first published studies the authors reported a modest induction of grass pollen allergen extract‐specific IgG^75^ and a reduction in allergen‐induced conjunctival inflammation as determined by conjunctival provocation test (CPT).^76^, ^77^

Although a hydrolyzed grass pollen extract was used with the goal to reduce allergenic activity of the vaccine, an immediate‐type grade IV reaction requiring epinephrine was observed in the large randomized, multicenter, double‐blind, placebo‐controlled trial.^77^ In a selected patients population it appeared that the hydrolyzed *Lolium perenne* grass pollen vaccine is clinically effective^75^ but unfortunately the clinical results of the phase III trial were disappointing.^78^

**FIGURE 1:**
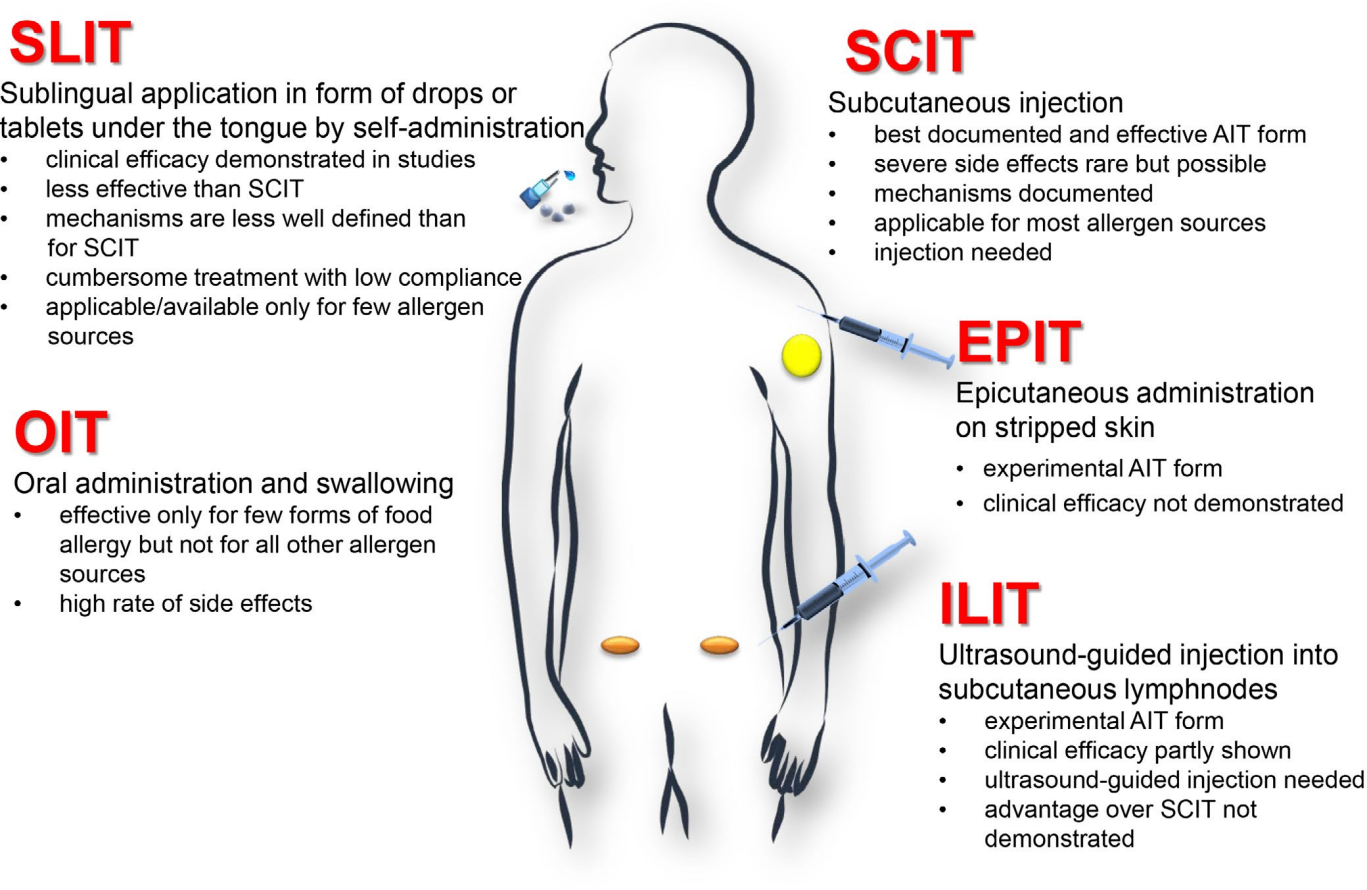
Routes for administration of AIT. Shown are different routes of administration for AIT and their features are mentioned

Besides the recent approach of using hydrolyzed allergen extracts, several other attempts have been made to improve allergen extract‐based AIT approaches. One major focus has been to test different routes of administration (Figure 1). Besides subcutaneous AIT (SCIT) for which the underlying mechanisms are established best and which is used for the longest time, actually since 1911, also other routes of administration have been considered.

### 3.2 Sublingual immunotherapy

Without doubts the greatest effort in terms of investment, development, and clinical trials has been dedicated to sublingual allergen administration in the last 30 years which has been summarized in a recent review article in a comprehensive way.^79^ The first attempts of sublingual immunotherapy go back to the study of Scadding & Brostoff in 1986^55^ and a first randomized controlled trial was published in 1998 by Passalacqua and colleagues.^56^ Sublingual immunotherapy (SLIT) was thought to reduce side effects and thus to increase the ease of use of AIT by self‐medication. Many controlled studies indicate that SLIT is clinically effective but until now the underlying mechanism is not fully understood because the treatment induces only very modest allergen‐specific IgG responses and paradoxically increases allergen‐specific IgE responses strongly.^80^ Surprisingly, the ability of SLIT to reduce immediate allergic symptoms as measured by controlled in vivo provocation testing such as SPT has not been studied extensively. Experts have pointed out that it is difficult to calculate the placebo effect in AIT in general^81^ and specifically in SLIT^82^ even in placebo‐controlled studies because it is difficult to blind for local reactions at the application site. Nevertheless, SLIT approaches have been evaluated in multiple clinical studies and clinical efficacy has been demonstrated in several phase III studies.^79^ However, real‐life‐compliance for SLIT is much lower than that for SCIT.^83^ After 3 years of treatment less than 7% of SLIT patients adhere to treatment. Furthermore, for many important allergen sources, such as venoms, food allergy, and many respiratory allergen sources, such as cat dander and molds, no effective SLIT treatments are available.

### 3.3 Oral immunotherapy

Another alternative approach to SCIT is oral immunotherapy (OIT). After the early OIT reports^54^ this approach has been tried for respiratory allergen sources but was found not to be effective.^84^, ^85^ Since beneficial effects were observed only with encapsulated respiratory allergen extracts^86^ it appears that OIT will not be effective for allergens which are easily digested in the gastrointestinal tract such as for the majority of respiratory allergens. It is therefore not a surprise that OIT is practiced mainly for certain food allergen sources which contain digestion‐resistant allergens such as milk, egg, peanuts, and to some extent wheat, whereas it is not used at all for other allergen sources. Clinical OIT studies basically show that beneficial effects are associated with the induction of allergen‐specific IgG antibodies which can block the IgE‐allergen interaction similar as for SCIT.^87^ In addition, OIT was reported to induce also alterations of cellular immune responses which may lead to clinical oral tolerance. However, despite showing clinical efficacy, OIT can induce severe side effects and there is currently only one registered OIT vaccine available which has undergone evaluation in large clinical trials. In this context, a recent review summarizing experience with OIT for peanut allergy^88^ concluded that “available oral peanut allergy immunotherapy regimens considerably increase allergic and anaphylactic reactions over avoidance or placebo, despite effectively inducing desensitization”.

### 3.4 Intralymphatic immunotherapy

As another alternative for SCIT, it has been suggested to perform intralymphatic administration in the form of intralymphatic immunotherapy (ILIT). A recent review by Senti and colleagues^89^ provides an excellent overview about ILIT. The basic idea behind ILIT is that the lymph nodes are rich in immune cells and direct exposure of allergen will lead to the induction of a faster and stronger protective IgG response and immunomodulation than SCIT.^90^ This assumption sounds logic but interestingly no detailed studies have been performed which have compared immunological and clinical responses of ILIT and SCIT with the same allergen vaccines side by side. It is therefore difficult to say if ILIT indeed induces faster and stronger immunological and clinical effects than SCIT. The safety profile of ILIT was found to be acceptable but a disadvantage of the treatment is that is requires ultrasound guidance to deliver the vaccine into the lymph nodes which makes it quite cumbersome. As compared with other routes relatively few studies have been performed so far mainly with allergen extracts and few with defined purified allergen derivatives.^57^

### 3.5 Epicutaneous immunotherapy

More than 10 years ago the first studies appeared, suggesting epicutaneous allergen administration for epicutaneous immunotherapy (EPIT).^91^, ^92^ An overview of experience gained in EPIT trials is summarized in a very recent review article.^93^ This review concludes that “EPIT might induce desensitization in peanut allergy and an increased risk of local adverse events (AEs). These findings should be interpreted with caution owing to the limited study and heterogeneity. More data in the older (children ≥ 12 years and adults) and other allergic diseases are needed”.

In fact, a detailed study of systemic allergen‐specific antibody, T‐cell, and cytokine responses after epicutaneous allergen administration has indicated that it induces only very modest systemic IgG increases and a detectable activation of allergen‐specific T‐cell responses.^94^ In fact, the analysis of systemic peanut allergen‐specific IgG responses in the course of EPIT has confirmed these findings. A very low induction of IgG responses only to certain peanut allergens was noted.^95^ The idea behind EPIT is that allergen administration via nonvascularized epidermis would induce less systemic side effects but relevant local AEs were reported. EPIT is a needle‐free treatment and hence was considered to be especially suitable for children. The treatment uses high doses of the allergen and, although showing some improvement in seasonal symptoms, does not demonstrate significant benefits in terms of local side effects when compared with SCIT.^96^ In a multicenter double‐blind, placebo‐controlled trial, 74 participants were randomized using two different doses of peanut patch and showed modest but statistically significant treatment effects compared with placebo when measured by oral food challenge. This was more evident in the patients younger than 11 years.^97^ Another recent study conducted in peanut allergic children reported positive effects.^98^ However, the primary endpoint (ie, percentage difference in responders between the active and placebo on eliciting dose determined by food challenges at baseline and 12 months thereafter) with a threshold of 15% or more on the lower bound of a 95% confidence interval around responder rate difference was not reached in this study. Regarding peanut allergy a recent review concluded that EPIT has no important advantages and benefits as compared with other approaches such as OIT or SLIT and states “There are no perfect treatments for peanut allergy and OIT, EPIT, and SLIT each has its unique pros and cons”.^99^

### 3.6 Short comparison of different routes for AIT

A short comparison of AIT routes as displayed in Figure 1 is given here. Without any doubt, SCIT is the AIT form for which mechanisms are documented best because it is in use since the discovery of AIT in 1911. No other AIT form as SCIT has been tested so extensively in clinical studies. SCIT can be used for most allergen sources (eg, respiratory allergen sources, venoms, etc). By using molecular approaches SCIT may become applicable also for food allergy as demonstrated for fish allergy (https://clinicaltrials.gov/ NCT02382718; NCT02017626). Much has been invested by companies to develop SLIT in the last 30 years but clinical efficacy of SLIT is usually lower than that of SCIT, treatment schedules are very cumbersome. Accordingly patients’ compliance is much lower than for SCIT.^83^ The mechanisms of SLIT are also less well defined than for SCIT. SLIT cannot be used for all allergen sources; in particular, not for food allergy and venom allergy and for many respiratory allergen sources no SLIT treatments are available. OIT is effective for few forms of food allergy (ie, digestion‐resistant food allergens) but at the expense of severe side effects. OIT cannot be used for respiratory allergens or venom allergens. EPIT is a relatively new and experimental form of AIT for which clinical efficacy has not been firmly established. Likewise, ILIT is a relatively new form of AIT for which clinical efficacy has not been established and potential advantages over SCIT have not been investigated in comparative studies.

### 3.7 Clinical experience with new adjuvants

It thus appears that the use of alternative routes cannot overcome the limitations set by the quality of natural allergen extracts. However, attempts were also made to improve SCIT by using alternative adjuvants. The type‐B immunostimulatory phosphorothioate oligodeoxynucleotide 5′‐TGACTGTAACGTTCGAGATGA (ODN‐1018) was tested in ragweed‐stimulated PBMC responses. It promoted Th1 cytokine and IL‐12 responses at the expense of Th2 cytokine production which could be further enhanced by conjugation of ODN‐1018 with the major ragweed allergen, Amb a 1.^100^ A functionally similar type B phosphorothioate oligodeoxynucleotide (ODN‐2006) activated human pDCs through TLR9 and induced regulatory T cells.^101^ A randomized, double‐blind, placebo‐controlled, phase 2 trial examined the effects of six weekly injections of the ODN‐1018‐Amb a 1 conjugate on ragweed‐induced allergic rhinitis. Treatment was associated with improvement in peak season nasal and quality of life symptom scores.^51^ However, this combination failed at the phase III trial. Another adjuvant is a derivative of monophosphoryl lipid A (MPL) from bacterial lipopolysaccharide. The structurally modified MPL acts through TLR4 to induce IL‐12 production and promote Th1 responses to allergen by human PBMC.^102^ In a randomized, double‐blind, placebo‐controlled study, a vaccine containing MPL and tyrosine‐absorbed glutaraldehyde‐modified extracts of grass pollen (Pollinex Quattro) reduced hay fever symptoms and increased allergen‐specific IgG when administered as four pre‐ and co‐seasonal injections.^103^

Monophosphoryl lipid A (MPL‐A) was also studied extensively as adjuvant in pollen SCIT. Asthmatic children were tested with one course of MPL‐SCIT and showed a reduction in bronchial hyper‐reactivity upon allergen provocation after treatment.^104^ However, a big phase III study performed in 1028 individuals, did not confirm previously achieved success with l‐tyrosine‐adsorbed, glutaraldehyde‐modified grass pollen extract containing MPL (Grass MATA MPL). Only 13% of improvement was reached after four subcutaneous injections in the treated group compared with placebo (Identifier: NCT00414141).

## 4 MOLECULAR IMMUNOTHERAPY FOR FUTURE TREATMENT AND ALLERGEN‐SPECIFIC PREVENTION

The era of molecular allergology started with the isolation of the DNA‐encoding major allergens more than 30 years ago.^105^, ^106^, ^107^, ^108^ Already shortly after the expression of the first recombinant allergen molecules, first studies demonstrated that molecular allergy diagnosis with purified recombinant allergens is possible and can replace allergen extract‐based testing.^109^, ^110^ Soon thereafter recombinant allergen molecules became available in commonly used in vitro allergy test systems worldwide.^111^ A major step in molecular allergy diagnosis was the development of chips containing micro‐arrayed allergen molecules for multiplex allergy diagnosis with small amounts of serum.^112^ Today molecular allergy diagnosis has revolutionized allergy diagnosis.^113^, ^114^ Besides resolving complicated sensitization patterns, molecular allergy diagnosis is of particular importance and useful for the prescription of AIT, the selection of patients for AIT, and the monitoring of AIT effects as well as a surrogate marker for the success of AIT.

### 4.1 Molecular allergy diagnosis for prescription and monitoring of AIT

The concept of using allergen molecules as diagnostic tests for prescription and monitoring of AIT was presented the first time at the Meeting of the European Academy for Allergy and Clinical Immunology (EAACI) held 2002 in Naples, Italy.^115^ Shortly thereafter micro‐arrayed allergens were developed and proposed for the same purpose.^112^ The fundamental idea behind the use of allergen molecules was that allergen sources contain source‐specific allergens and cross‐reactive allergens. IgE reactivity to the source‐specific allergens serves as an indicator that the patient is genuinely sensitized against a particular allergen source. On the other hand IgE reactivity with highly cross‐reactive allergens was considered helpful to discriminate genuine sensitization from cross‐sensitization. Thus, the availability of molecular testing was suggested to assist in the selection of correct allergy vaccines. In the meantime this concept was found to be very helpful for the correct prescription of AIT as has been demonstrated in several clinical studies thereafter.^116^, ^117^, ^118^, ^119^, ^120^, ^121^

Thus, molecular allergy diagnosis is well established as a companion diagnosis to guide the prescription of AIT and for the monitoring of the effects of AIT.^122^ It is currently used as companion diagnosis for allergen extract‐based AIT but will be even more useful for molecular AIT approaches. Furthermore, molecular allergen reactivity profiles as determined for different countries and populations are important for the selection of allergen molecules to be incorporated into new molecular AIT vaccines.^7^, ^123^, ^124^

### 4.2 Molecular AIT approaches

**FIGURE 2:**
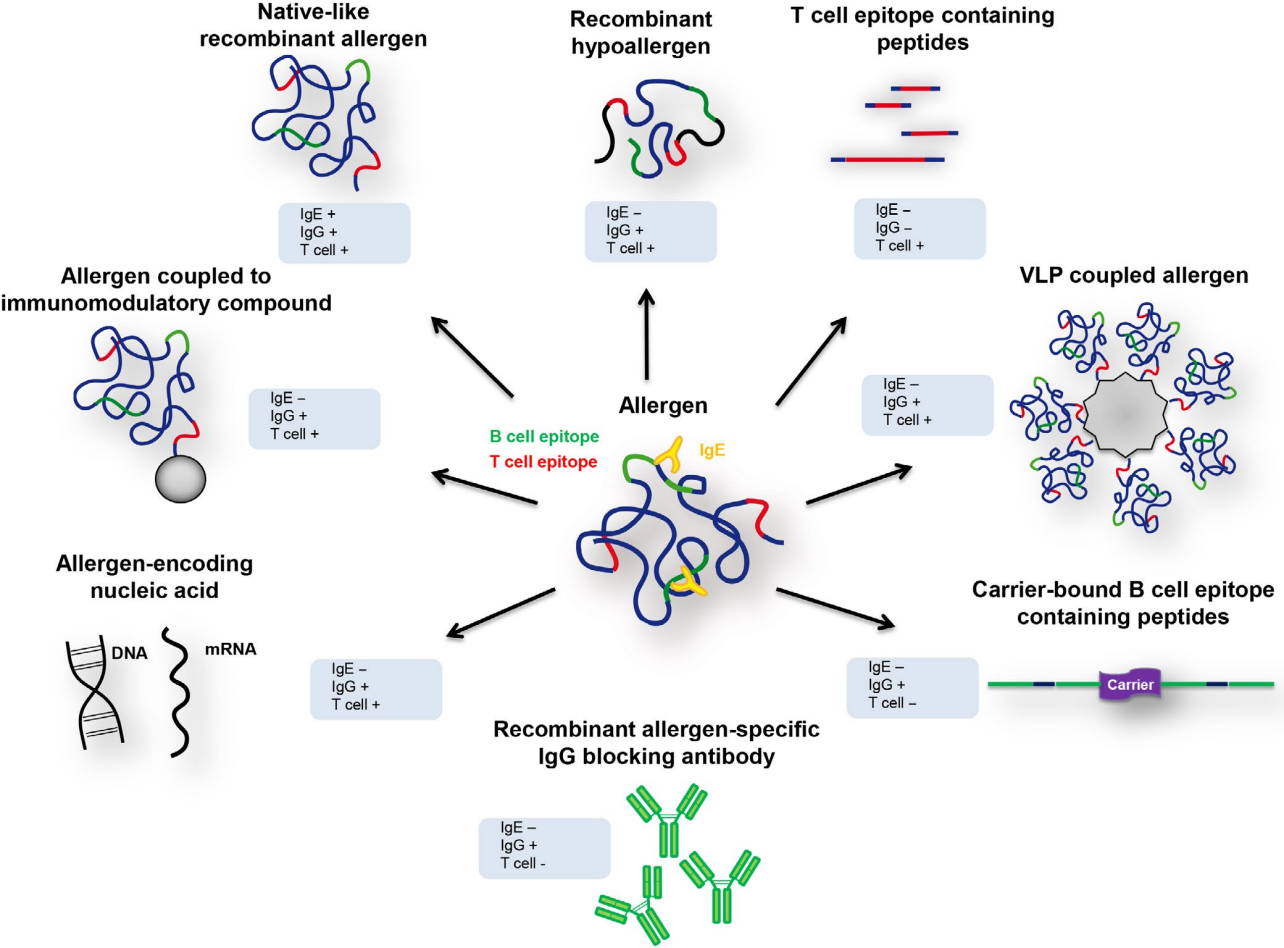
Molecular forms of AIT. Based on the knowledge of the DNA sequence and molecular structure of the disease‐causing allergens different molecular AIT strategies have been developed. The different strategies comprise native‐like recombinant allergens, recombinant hypoallergens, T‐cell epitope‐containing peptides, VLP‐coupled allergens, carrier‐bound B‐cell epitope‐containing peptides, recombinant allergen‐specific IgG blocking antibodies, allergen‐encoding nucleic acids, and allergens coupled to immunomodulatory compounds. The blue boxes inform about IgE‐ and T‐cell reactivity of each component and its ability to IgG blocking antibody activity

The different molecular approaches for AIT are displayed in Figure 2. In fact, everybody would have expected that the use of native‐like recombinant allergens mimicking the immunological properties of the natural allergen molecules would have been the first approach for molecular AIT. However, the molecular era of AIT started with attempts to treat patients with short, non‐IgE‐reactive, allergen‐derived T‐cell epitope‐containing peptides followed by recombinant hypoallergenic allergen derivatives with reduced IgE reactivity before first AIT trials were conducted with native‐like recombinant allergen molecules. The reason for this may have been that investigators were keen to improve immediately two aspects of AIT, specificity by using defined molecules and safety by using allergen derivatives which were designed to have reduced allergenic activity. Figure 2 provides an overview of different molecular AIT approaches of which many have already been considered in an perspective article published already in 1999.^125^ In the meantime, these approaches have been evaluated in clinical studies and an overview of the state of the art of molecular AIT approaches is provided in a recent review article.^25^ This recent overview article provides an overview of the different molecular AIT trials regarding targets and trial designs. A brief summary of each of the molecular AIT approaches and potential ways forward follow below.

### 4.3 T‐cell epitope‐containing peptides

Allergen‐derived T‐cell epitope‐containing peptides are synthetic peptides comprising T‐cell epitopes of allergen lacking IgE reactivity (Figure 2). The goal of injecting such peptides into patients was to induce T‐cell tolerance and it was hoped that this would reduce allergen‐specific IgE production and allergen‐induced inflammation.^126^ The approach was started already soon after the first allergen DNAs were isolated using Fel d 1, the major cat allergen as a model.^44^, ^45^ However, the first trials were not successful and even when the approach was refined more recently, peptide‐based AIT was not effective. In fact, in a phase III trial involving more than 1000 patients a huge placebo effect was observed and active treatment did not achieve improvement over placebo.^127^ The T‐cell epitope‐containing peptides are relatively short and, accordingly, this treatment did not induce allergen‐specific protective IgG antibodies which may be one reason for the failure of this approach. Although the T‐cell epitope‐based approach was not successful for the treatment of patients with allergy, it is quite possible that this strategy may be useful for the induction of T‐cell tolerance in a preventive approach but this has not yet been studied in clinical trials.

### 4.4 Recombinant and synthetic hypoallergens

After the T‐cell epitope‐containing peptides, recombinant hypoallergenic allergen derivatives were the first to be evaluated in clinical AIT studies in patients.^62^, ^128^, ^129^ Recombinant hypoallergenic allergen derivatives are derived from recombinant allergens which are rendered hypoallergenic by genetic engineering or chemical modification.^130^ Different modes of genetic engineering have been developed which include fragmentation, oligomerization, mutation, and reassembly of sequences with the goal to reduce IgE reactivity which usually is obtained by altering or destroying the structural allergen fold. This can be also achieved with immunogenic synthetic peptides.^131^, ^132^ The hypoallergens are hence characterized by reduced IgE reactivity. They do not elicit immediate side effects, but upon immunization, induce allergen‐specific IgG antibodies to a various extent (Figure 2). Allergen‐specific T‐cell epitopes remain largely preserved in these molecules and hence may give rise to late‐phase, T‐cell–mediated side effects. Available proof of principle AIT studies in patients demonstrate that hypoallergens are clinically effective.^25^ Furthermore, preclinical studies demonstrate that hypoallergens induce blocking IgG antibodies and can be developed for many respiratory, venom, and also food allergens.^133^, ^134^, ^135^, ^136^, ^137^ In fact, recent results of a I/IIa phase double‐blind, placebo‐controlled clinical study (Identifier: NCT02017626) involving 15 fish allergic patients demonstrated that immunization with a Cyp c 1 mutant designed for subcutaneous AIT induces production of IgG‐specific antibodies and showed low levels of side effects.

A further development of recombinant hypoallergens are the carrier‐bound B‐cell epitope‐containing peptides in which the presence of allergen‐specific T‐cell epitopes has been reduced to further decrease allergenic activity and thus to increase safety.^53^, ^138^ The use of carrier molecules in the latter derivatives facilitates their production and increases their immunogenicity and ability to induce blocking IgG antibody responses. A clinical efficacy of approximately 25% of improvement of the combined symptom medication score over placebo has been achieved with this approach (clinical study identifier: NCT02643641).^139^

### 4.5 Native‐like recombinant allergens

Soon after the first recombinant allergens have been produced it was also demonstrated by in vitro experiments that recombinant allergens resemble the features of their natural counterparts and account for a high proportion of epitopes/allergens present in natural allergen extracts (eg, IgE antibodies to recombinant pollen allergens).^140^, ^141^ Two important AIT studies have demonstrated equivalence of recombinant allergens with complex allergen extracts. The study by Jutel *et al* showed that a mix of recombinant grass pollen allergens for SCIT is clinically effective^46^ and the elegant study by Pauli *et al* proved that SCIT with recombinant Bet v 1 was equivalent to SCIT with natural birch pollen extract and purified natural Bet v 1 but had additional advantages by inducing higher levels of blocking IgG and avoided sensitization to minor allergens.^47^ The importance of the two studies with native‐like recombinant allergens is that they demonstrate the equivalence and even advantages of recombinant allergens over natural allergen extracts.

Native‐like recombinant allergens could therefore be used instead of natural allergen extracts and would offer the advantage to be well‐defined and to contain all relevant epitopes. SCIT with these molecules induces allergen‐specific blocking IgG. However, like natural allergens they induce immediate‐type and late‐phase side effects because of preserved IgE reactivity and T‐cell epitopes. Accordingly, SCIT with native‐like recombinant allergens requires the same cumbersome up‐dosing schedules and multiple maintenance injections as SCIT with natural allergen extracts. However, the advantage over natural allergen extracts is the high quality of the vaccine because allergens can be produced at low cost in a defined and reproducible manner. In this context, also a recent successful SCIT study using purified natural major *Alternaria* allergen Alt a 1 should be mentioned.^142^ As recombinant Alt a 1 was found to be equivalent with natural Alt a 1 one may assume that this could have been equally achieved with purified rAlt a 1.

### 4.6 Allergens coupled to immunomodulatory compounds

CpG oligodeoxynucleotides were found to be useful as adjuvants to induce Th1 immune responses and their immunomodulatory activity in a murine model of asthma was demonstrated.^143^ Researchers from the company Dynavax which had pursued nucleic acid‐based AIT approaches found that coupling of such immune‐stimulatory DNA to the major ragweed allergen Amb a 1 enhanced its immunogenicity and at the same time reduced its allergenicity.^144^ Based on this finding a SCIT trial was conducted with this vaccine showing that it induced allergen‐specific blocking antibody responses and led to a reduction in allergen‐specific IgE levels.^51^ The vaccine also showed beneficial clinical effects but the approach was not further pursued because the outcome of subsequent studies was less favorable and the production of the vaccine by chemical coupling turned out to be challenging.

### 4.7 Virus‐like particle‐coupled allergens

The first generation of virus‐like particle‐coupled allergens followed a similar principle as described for allergens coupled to immunomodulatory sequences. Allergen molecules were coupled chemically ^145^, ^146^, ^147^ and also by specific linker systems to virus‐like particles produced by recombinant expression.^148^ As a result, a reduced allergenic activity of the vaccines and good immunogenicity were noted. A major hurdle for the virus‐like particle coupled allergens for further clinical use is that it is very difficult to produce these vaccines in a reproducible manner due to the coupling process which is difficult to control. It is quite possible that the reduced allergenic activity of these vaccines is achieved not only by “hiding” of IgE epitopes by the VLPs but also by the formation of large oligomers as was observed for a recombinant Bet v 1 trimer^149^ and a hybrid molecule consisting of Bet v 1 and the grass pollen allergen Phl p 5.^150^ Large oligomers seem to function as poor cross‐linkers of mast cell and basophil‐bound IgE due to unfavorable steric presentation of IgE epitopes.

A more sophisticated approach of engineering virus‐like nanoparticles (VNP) containing shielded or unshielded allergens was recently reported. In this approach, allergen‐encoding cDNA is fused to virus‐encoding DNA (Matrix protein, p15MA)^151^ or to a glycosyl‐phosphatidyl inositol anchor acceptor sequence^152^ to be expressed either inside or outside of VNPs, respectively. Encasement of full‐length allergens within VNPs should ensure that such VNP preparations are nonanaphylactogenic when applied to already sensitized individuals.^153^ In a preclinical model of mugwort pollen allergy^154^ such particles were successfully used for prophylactic vaccination.^155^ However, there is so far no experience with virus‐like particles in clinical AIT studies in patients, although one study performed in nonallergic subjects showed the induction of allergen‐specific IgG antibodies.^156^

### 4.8 Allergen‐encoding nucleic acids

More than 20 years ago, two experimental studies were published, indicating that it is possible to vaccinate with allergen‐encoding DNA to obtain an allergen‐specific Th1 response and allergen‐specific IgG responses in murine models.^48^, ^49^ This finding was important because it indicated that DNA vaccination for AIT may be possible.^157^ Major challenges of this approach were to prevent uncontrolled release of allergen in the vaccinated person and thus the risk of allergic side effects. To this end DNA vaccination with DNA coding for hypoallergens and RNA vaccination which has a reduced risk of generating relevant allergen amounts in the tissues were considered.^158^ A first clinical study reporting DNA vaccination has been published, ^50^ but no information is available if this strategy induces a protective immune response.

### 4.9 Carrier‐bound B‐cell epitope‐containing peptides

Carrier‐bound B‐cell epitope‐containing peptides represent a further development of recombinant hypoallergens.^138^, ^159^ The currently most advanced platform uses hepatitis B–derived PreS protein as a carrier protein to which nonallergenic peptides derived from the IgE‐binding sites of allergens are fused. The vaccines are made as recombinant fusion proteins by expression in *Escherichia coli* by a well‐controlled procedure delivering large amounts of the fusion proteins in consistent quality.^160^, ^161^, ^162^, ^163^ The hypoallergens are characterized by lack of IgE reactivity and hence do not induce any immediate side effects but blocking IgG antibodies upon immunization.^164^ Allergen‐specific T‐cell epitopes are largely replaced by the carrier molecule which reduces late‐phase, T‐cell–mediated side effects. Importantly, the grass pollen allergy vaccine BM32 which has been constructed according to this principle has been tested in several clinical trials and was found to be safe and to induce beneficial clinical effects even in double‐blind, placebo‐controlled multicenter field studies.^52^, ^53^, ^137^, ^165^ BM32 was found to induce continuously growing allergen‐specific IgG_4_ response with little allergen‐specific T‐cell activation.^22^ BM32, unlike most other AIT vaccines does not induce or boost allergen‐specific IgE responses but rather blunts seasonally boosts of IgE production. Another unexpected but important finding was that antibodies induced against the hepatitis B–derived PreS protein protected against hepatitis B infection in vitro*.* ^166^ Therefore, one of the components of BM32 (ie, BM325) is currently tested in a clinical trial for its ability to induce protective immune responses against hepatitis B, in particular in nonresponders to current only S protein–based vaccines and for its value as a therapeutic hepatitis B vaccine (ClinicalTrials.gov: NCT03625934). Besides the grass pollen allergy vaccine, similar peptide carrier vaccines have been developed for several other respiratory allergen sources and the technology may be also applicable for the production of AIT vaccines against venom and food allergens. BM32 has undergone three phase II studies and a phase III study is currently planned to make the vaccine ready for registration.

### 4.10 Recombinant allergen‐specific IgG blocking antibodies

The demonstration of Dunbar that allergen‐specific antisera can prevent allergic reactions in vivo^28^ and the study by Cooke showing that AIT‐induced IgG can suppress allergen‐induced immediate‐type skin reactions^33^ indicated that the induction of allergen‐specific IgG antibodies is a major mechanism of AIT. However, in order to obtain defined vaccines for passive immunization, human allergen‐specific blocking antibodies are needed. More than 20 years ago the first publications appeared in which such allergen‐specific antibodies were reported. One study reported human monoclonal IgA antibodies specific for the major ragweed allergen, Amb a 1,^167^ and shortly thereafter, a highly potent human IgG antibody (ie, BAB1) which could prevent allergic patients’ IgE binding to the major birch pollen allergen Bet v 1 was isolated.^168^ Of note, BAB1 also inhibited Bet v 1–induced basophil activation. In order to obtain IgG antibodies which are equivalent to IgE antibodies, a combinatorial library was constructed from peripheral blood mononuclear cells of a grass pollen allergic patient and for the first time human allergen‐specific IgE Fabs were isolated.^169^ One of these IgE Fabs which was specific for the major grass pollen allergen Phl p 2 could be converted into a complete recombinant human IgG antibody which blocked Phl p 2–induced basophil degranulation and hence had therapeutic potential.^170^ In order to obtain recombinant human allergen‐specific IgG which can already block the entry of allergens through the respiratory epithelium, bi‐specific antibodies were created. In fact, it could be demonstrated that one can immobilize allergen‐specific blocking IgG antibodies via ICAM1‐specific IgG onto the respiratory epithelium and thus prevent allergen penetration^171^ which opens the possibility for topical treatment with blocking antibodies.

The ultimate demonstration for the efficacy of passive immunization with allergen‐specific recombinant antibodies for therapy of allergy was recently reported. Immunization with two monoclonal IgG_4_ antibodies specific for the major cat allergen, Fel d 1, protected against cat allergy in a clinical study conducted in an exposure chamber.^36^, ^37^ The concept of passive immunization with allergen‐specific recombinant IgG antibodies is intriguing and is certainly a possible approach for allergen sources which contain mainly one major allergen which can be blocked with one or few monoclonal antibodies. This approach will be particularly useful for seasonal allergies because one preseasonal immunization may be sufficient to protect the patient during seasonal allergen exposure. However, it will be very difficult, if not impossible to protect patients suffering from allergy caused by allergen sources containing several potent allergen components such as grass pollen allergy, house dust mite allergy, and peanut allergy to mention a few because this would require the production of multiple monoclonal antibodies. Conceptually, the demonstration that passive treatment with allergen‐specific IgG is effective for AIT is important because it teaches that one requirement for successful AIT vaccine is the induction of high titers of allergen‐specific blocking IgG antibodies. One can thus imagine for the future that recombinant active allergen‐specific AIT approaches as well as passive immunization strategies will be available and can be given depending on the need of the patient, similar to active and passive immunization for prevention of infectious diseases.

### 4.11 Cell‐based therapy

Much has been learned about the robust induction of immunological tolerance from the field of transplantation immunology.^172^, ^173^, ^174^, ^175^ Everybody remembers the classical experiments demonstrating that the transfer of hematopoietic stem cells from one mouse strain to another strain with different MHC background early in life induced tolerance and prevented subsequent transplant rejection.^176^ Using the approach of transducing hematopoietic murine stem cells to express allergens on their surface, Baranyi and colleagues succeeded to demonstrate that mice that had received such allergen‐expressing cells could not be sensitized against the corresponding allergen. Even when a protocol of allergic sensitization by aluminum‐hydroxide adsorbed allergens was used it was not possible to induce allergen‐specific T cell, antibody (of any isotype, including IgE), or allergic immune responses indicating that life‐long robust tolerance was obtained, likely relying on mechanisms of central tolerance rather than peripheral regulation.^177^ This concept still requires immunomodulatory treatment, uses a protocol for cell transduction which may be potentially hazardous and needs to be applied early in life, most likely immediately after birth. However, it shows that robust and life‐long allergen‐specific immunological tolerance can be achieved by a cell‐based treatment. Recently the requirements for short‐course immunomodulatory treatment at the time of cell transfer could be markedly reduced.^175^, ^178^ However, the cell‐based allergen‐specific prevention approach is highly experimental but warrants further investigation in clinical trials once the major safety hurdles can be overcome.

## 5 FUTURE NEEDS IN AIT

In Table 2 we have summarized different indications for which AIT can be used together with the current status for these indications and the yet unmet needs. Regarding the treatment of allergic rhinoconjunctivitis and of mild asthma, only few SCIT and SLIT vaccines for few pollen allergen sources and house dust mite (HDM) allergy have been produced utilizing a Good Manufacturing Process (GMP) and have been evaluated in extensive clinical trials so that registered products are available.^19^ The vast majority of SCIT and SLIT vaccines for this indication do not meet these criteria and are available only as named patient's products without documented evidence for efficacy. Clearly, the unmet need here is to have GMP‐manufactured vaccines with documentation for efficacy by clinical trials for all relevant respiratory allergen sources. For severe asthma, atopic dermatitis, and food allergy there is evidence that AIT can be effective.^179^, ^180^, ^181^ but there are basically only experimental treatments available. Here, the need is to define what AIT strategy is best suited and GMP‐manufactured vaccines with documentation for efficacy by trials are needed. For the treatment of venom allergy (ie, bee and wasp allergy), there are SCIT vaccines available on a named patient´s basis but GMP‐manufactured vaccines with proven clinical efficacy as demonstrated by clinical trials are missing.^182^

**TABLE 2:** Indications for AIT, current status, unmet needs

Indication	Current status	Unmet need
Treatment		
Treatment of rhinoconjunctivitis and mild asthma	GMP‐manufactured vaccines (SCIT, SLIT) evaluated in clinical trials available only for few pollen allergens sources and HDM SCIT and SLIT vaccines without documentation available as named patient's products	GMP‐manufactured vaccines evaluated in clinical trials for all respiratory allergen sources
Treatment of moderate‐to‐severe asthma	Evidence for AIT efficacy from trials and experience but no AIT vaccines available	Clinical trials to determine best strategies GMP‐manufactured vaccines evaluated in trials for important respiratory allergen sources
Treatment of atopic dermatitis	Evidence for AIT efficacy from trials and experience but no AIT vaccines available	Clinical trials to determine best strategies GMP‐manufactured vaccines evaluated in trials for important allergen sources causing AD
Treatment of food allergy	Experimental OIT vaccines for few allergen sources but no GMP‐manufactured vaccines evaluated in clinical trials available	Clinical trials to determine best strategies GMP‐manufactured vaccines evaluated in trials for important food allergen sources
Treatment of anaphylaxis (venoms)	SCIT vaccines without documentation available as named patient's products but no GMP‐manufactured AIT vaccines evaluated in clinical trials available	GMP‐manufactured evaluated in clinical trials for bee and wasp venom allergy
Secondary prevention		
Transition from rhinitis to asthma	Evidence for AIT efficacy from trials and experience but no AIT vaccines available	Clinical trials to determine best strategies GMP‐manufactured vaccines evaluated in trials
Transition of silent sensitization to symptomatic allergy	So far no evidence from clinical trials	Clinical trials to determine feasibility Identification of feasible strategies GMP‐manufactured vaccines evaluated in trials
Primary prevention		
(ie, prevention of IgE sensitization)	So far no evidence from clinical trials	Clinical trials to determine feasibility Identification of feasible strategies GMP‐manufactured vaccines evaluated in trials

Abbreviations: AIT, Allergen‐specific immunotherapy; GMP, Good Manufacturing Practice; IgE, Immunoglobulin E; SCIT, Subcutaneous immunotherapy; SLIT, Sublingual immunotherapy.

While there is evidence from a few clinical trials that AIT can be used in a secondary preventive approach to hold on the transition of rhinitis to asthma,^24^, ^60^, ^183^ no GMP‐manufactured vaccines for which this effect has been demonstrated in extensive clinical trials is available. Accordingly, there is a huge unmet need for this important indication.

It is quite conceivable that AIT can be used to prevent the transition of clinically silent IgE sensitization to the development of symptomatic allergy but this has not yet been demonstrated. The SLIT approaches which have been performed so far have been inconclusive^184^, ^185^ Preventive effects were noticed in the LEAP study when children were fed peanut allergens early, but this approach could not be extended to other forms of food allergy.^186^, ^187^ One would therefore rather consider using a SCIT approach for a preventive indication. There is a huge unmet need for clinical feasibility studies to identify suitable strategies and such secondary preventive vaccines would be very important to stop the allergy epidemics.

Finally, one may consider AIT approaches or new molecular allergen‐specific approaches for primary prevention. This means that the treatment will prevent the development of IgE sensitization. So far, there is no evidence that such an approach may be feasible but obviously there is a tremendous need for such strategies because they could potentially stop allergic sensitization.

It thus becomes obvious that there is a huge need to develop AIT for the above‐mentioned indications, first of all to be able to treat different allergic symptoms with effective AIT vaccines and, most importantly, to explore the use of AIT for preventive treatment. The field of AIT looks like a sleeping beauty waiting to be kissed awake. We think that a systematic further development of AIT can be performed only by using molecular approaches but not by allergen extract‐based approaches. First of all, molecular approaches are well defined and can be compared, whereas allergen extracts present a huge variability and a comparison of results is not possible. Second, everybody knows that mechanisms of treatment can be only elucidated using defined molecular models. This will never be possible with allergen extracts containing undefined mixtures of different allergens and additional unknown materials. The elucidation of mechanisms and consecutively the establishment of surrogate markers for efficacy will be very important to eventually reduce the need for clinical trials similar as for vaccines in infectious diseases. This would speed up AIT vaccine development. Furthermore, molecular approaches will allow optimizing the vaccine antigen itself by molecular design for the desired purpose in addition to the optimization of adjuvants and forms as well as schedules of administration.

### 5.1 Molecular AIT approaches for different allergic manifestations and new indications

AIT of moderate‐to‐severe asthma and AIT of food allergy are two indications which may benefit from molecular AIT strategies. Since severe side effects are a major disadvantage of OIT of food allergy and OIT can only be applied for digestion‐resistant food allergens, SCIT with recombinant hypoallergenic derivatives may be considered. In fact, it has been shown that SCIT with a hypoallergenic derivative of the major fish allergen, parvalbumin, was safe and a protective IgG response could be elicited.^133^, ^134^, ^135^ It is thus conceivable that one can develop SCIT approaches also for other forms of food allergy based on designing hypoallergenic food allergen molecules. Safety is a major issue for AIT of moderate‐to‐severe asthma. In fact, the approach of carrier‐bound B‐cell epitope‐containing peptides which is a second‐generation hypoallergenic approach characterized by a profound reduction in IgE‐mediated allergenic activity and a reduction in T‐cell–mediated side effects would seem particularly suitable for asthma treatment.^139^ In fact, the grass pollen allergy vaccine, BM32, which was developed by this approach showed a good safety profile in clinical studies and beneficial effects on asthma symptoms were noted in the field study. The carrier‐bound B‐cell epitope‐containing peptide approach is also unique among all AIT approaches as it does not boost IgE production.^137^ It should therefore be useful for secondary preventive AIT approaches such as AIT to prevent the progression of rhinitis toward asthma and for the prevention of the progression of clinically silent IgE sensitization toward symptomatic allergy. However, it has also been shown recently that vaccination of nonallergic subjects with recombinant hypoallergenic derivatives of the major birch pollen allergen, Bet v 1, was safe and induced allergen‐specific IgG antibodies which prevented allergic patients IgE binding to Bet v 1.^188^ In this study, the induction of allergen‐specific IgE could be detected but due to the massive production of allergen‐specific IgG no allergic sensitization occurred in the nonallergic subjects.

### 5.2 Molecular AIT approaches for allergen‐specific prevention

**FIGURE 3:**
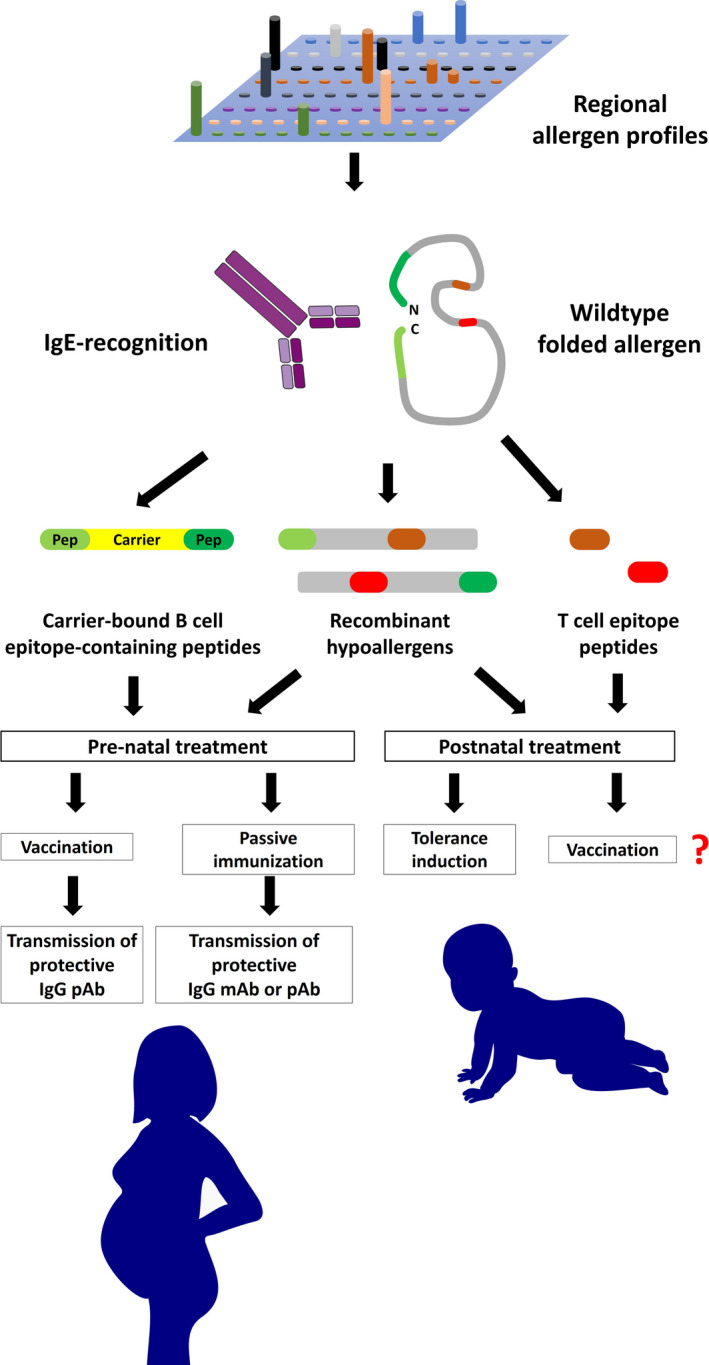
Toward allergen‐specific prevention of allergy. Using chips containing micro‐arrayed allergen molecules (top), it is possible to identify the clinically relevant allergen molecules for a given population. According to these profiles IgE‐ and T‐cell epitopes of these allergens can be mapped and used for the development of molecular approaches for allergen‐specific prevention (middle part). Carrier‐bound B‐cell epitope‐based peptides and recombinant hypoallergens may be used for prenatal vaccination of mothers to transmit blocking antibodies to the off‐spring or for early postnatal vaccination (Bottom). The administration of allergen‐specific blocking antibodies by passive immunization of mothers may also be considered for primary prevention. T‐cell epitope‐containing peptides may be used for early postnatal tolerance induction

Figure 3 provides an overview for allergen‐specific approaches for primary prevention of allergy. A major prerequisite for molecular approaches of primary allergy prevention is the knowledge of the molecular sensitization profiles of the target population. The molecular sensitization profiles can be easily established using micro‐arrayed allergen molecules.^7^, ^189^ According to the IgE reactivity profiles determined for a population, the most relevant allergen molecules can be selected according to prevalence of IgE reactivity and allergenic activity for the formulation of the preventive vaccines. It is clear that such a vaccine will not contain all molecules but data from birth cohort analyses have shown that one can define a reasonable number of important allergens.^124^ For active vaccination SCIT approaches based on hypoallergenic allergen molecules such as recombinant hypoallergens and carrier‐bound B‐cell epitope‐containing peptides may be considered as best approaches because these molecules exhibit low allergenic activity and induce allergen‐specific IgG antibodies. Besides early postnatal vaccination one may also consider to build up high levels of allergen‐specific IgG in females reaching peak levels in the end of pregnancy because evidence was provided that high levels of allergen‐specific IgG transmitted from mothers to off‐springs may protect against allergic sensitization.^190^ In this context, also passive immunization with allergen‐specific IgG could be possible but it will be difficult to make allergen‐specific IgG antibodies for several different allergens. Another possibility for primary prevention of allergy could be early tolerance induction to suppress the development of allergen‐specific adaptive immune responses. This could be achieved by the administration of hematopoietic stem cells, eventually cord blood cells, which are transfected with allergens or allergen‐derived peptides.^174^ Alternatively, oral tolerance induction by feeding allergens or allergen‐derived T‐cell epitope‐containing peptides may be possible.^191^, ^192^ The latter approaches clearly involve ethical considerations and will require extensive research in experimental models, whereas the active vaccination approach of using hypoallergens could enter relatively soon clinical trials. These trials will have to show the safety and lack of allergic sensitization in nonallergic adults. Next, trials studying secondary prevention followed by trials investigating primary prevention in proof of principle studies using a frequently recognized allergen in a population with high sensitization rates toward this molecule can be conducted.

### 5.3 Concluding remarks

AIT has a history of almost 110 years. It represents a therapeutic vaccination against allergy and has potential also for allergen‐specific prevention. AIT is the only disease‐modifying treatment for allergy and has several other important advantages over pharmacological treatments and biological therapies such as high clinical efficacy, long‐lasting effects, and cost effectivity. However, AIT has also disadvantages such a side effects and cumbersome treatment schedules. Much has been learned about the mechanisms of AIT in numerous preclinical and clinical studies. With the availability of the disease‐causing allergen molecules, a series of molecular AIT strategies has been developed which may revolutionize traditional allergen extract‐based AIT to allow more patients to benefit from AIT and to develop preventive AIT forms.

## CONFLICT OF INTEREST

W. Pickl holds stocks of Biomay AG and receives honoraria from Novartis and Pfizer. Dr Wekerle reports personal fees from Astellas, personal fees from Chiesi, personal fees from Therakos, grants from TEVA, outside the submitted work. Rudolf Valenta has received research grants from Viravaxx (Vienna, Austria) and serves as consultant for this company. The other authors declare that they have no competing interests.
